# Nestin^+^ progenitor cells isolated from adult human sweat gland stroma promote reepithelialisation and may stimulate angiogenesis in wounded human skin ex vivo

**DOI:** 10.1007/s00403-019-01889-x

**Published:** 2019-02-23

**Authors:** Tian Liao, Janin Lehmann, Sabine Sternstein, Arzu Yay, Guoyou Zhang, Anna Emilia Matthießen, Sandra Schumann, Frank Siemers, Charli Kruse, Jennifer E. Hundt, Ewan A. Langan, Stephan Tiede, Ralf Paus

**Affiliations:** 1 Department of Head and Neck Surgery, Fudan University Shanghai Cancer Center, Shanghai, China; 2 Department of Oncology, Shanghai Medical College, Fudan University, 200032 Shanghai, China; 3 Monasterium Laboratory, Muenster, Germany; 4 Academic Management, German Sport University of Cologne, Cologne, Germany; 5 Department of Histology and Embryology, University of Erciyes, Kayseri, Turkey; 6 Department of Plastic and Reconstructive Surgery, Shanghai Ninth People’s Hospital, Shanghai Jiao Tong University School of Medicine, 200011 Shanghai, China; 7 Fraunhofer Research Institution for Marine Biotechnology and Cell Technology (EMB), Luebeck, Germany; 8 Department of Plastic and Hand Surgery, BG Klinikum Bergmannstrost, Halle, Germany; 9 Department of Dermatology, University of Luebeck, Luebeck, Germany; 10 Centre for Dermatology Research, NIHR Manchester Biomedical Research Centre, University of Manchester, Manchester, UK; 11 Department of Biochemistry, Children’s Hospital, University Medical Center Hamburg-Eppendorf, Hamburg, Germany; 12 Department of Dermatology and Cutaneous Surgery, University of Miami Miller School of Medicine, Miami, FL USA

**Keywords:** Nestin, Angiogenesis, CD31, Reepithelialisation, Wound healing, Organ culture

## Abstract

The combination of an aging population and an increasing prevalence of diseases associated with impaired-wound healing, including obesity, peripheral vascular disease and diabetes, is likely to result in a dramatic increase in the incidence and prevalence of chronic skin wounds. Indeed, systemic reviews are now not only trying to establish both the prevalence and the often under-estimated socio-economic costs of chronic skin wounds, but most importantly are addressing the impact that chronic wounds have on quality of life. Given the clear need for novel approaches to the management of chronic skin ulceration, ideally developed and tested in the human system in a manner that can be rapidly translated into clinical practice, we examined the effects of multipotent primary human nestin^+^ progenitor cells on human wound healing in an ex vivo model. Human sweat gland-derived nestin^+^ cells demonstrated the capacity to significantly promote two key wound healing parameters, i.e., both reepithelialisation and angiogenesis in experimentally wounded, organ-cultured human skin. The current data further support the use of full-thickness human skin wound-healing models ex vivo to pre-clinically test wound healing-promoting candidate agents. Whilst larger studies are required to substantiate a firm “proof-of-concept,” our preliminary studies encourage further efforts to systemically determine the potential of cell-based regenerative medicine strategies in general, and the use of skin appendage-associated human nestin^+^ cells in particular, as novel treatment strategies for chronic skin ulceration.

**Electronic supplementary material:**

The online version of this article (10.1007/s00403-019-01889-x) contains supplementary material, which is available to authorized users.

## Background

The management of chronic skin ulcers constitutes a major healthcare challenge [6, 16], which is exacerbated by the increasing prevalence of vascular perfusion disorders, diabetes mellitus and other conditions associated with impaired-wound healing. Therefore, novel treatment strategies for chronic skin wounds need to be urgently developed and tested; ideally in the human system. For safety, cost, and regulatory reasons, cell-based regenerative medicine approaches, with transplanted autologous adult progenitor cells, have long been viewed as a particularly promising skin-regeneration strategy in this context [1, 10, 18].

In the current pilot study, we have attempted to generate preclinical proof-of-principle for one such cell-based ulcer management strategy: administering multipotent primary, human nestin^+^ progenitor cells to experimentally wounded, organ-cultured full-thickness human skin, using a punch-within-a-punch design [12]. These wounded human skin fragments were transplanted with adult nestin^+^ progenitor cells derived from adult human sweat gland (eccrine and apocrine) stroma (nestin^+^-SGSCs), which had been isolated and characterized as previously described [9, 14, 15, 18]. These stromal cells contained 80% nestin^+^-SGSCs before in vitro transplantation, showed the expected multilineage differentiation capacity [14, 15] and improved vascularisation in vivo in a mouse model for dermal regeneration [5].

The current study builds on previous work that had demonstrated the presence of nestin^+^ pluripotent cells in murine hair follicles and reported their capacity to promote angiogenesis and nerve/spinal cord repair [2–4]. In human skin, these nestin^+^ cells are predominantly found in the stroma of skin appendages, namely of hair follicles (HFs) and, most prominently, of sweat glands [18]. Given that HFs themselves may influence wound healing, sweat gland stroma was selected to isolate nestin^+^ cells. We specifically asked whether the transplantation of adult human nestin^+^-SGSCs into the wound bed of adult human skin enhances epidermal regeneration and angiogenesis, two key components of skin wound healing [10].

## Materials and methods

Nestin^+^-SGSCs were isolated from excess axillary skin obtained during elective plastic surgery, while 4 mm skin punch biopsies (with a partial thickness central 2 mm punch biopsy, see Fig. S1) were derived from facelift surgery, after patient consent and formal ethical approval. Transplanted nestin^+^-SGSCs were labelled with nanoparticles to facilitate their visualization (Fig. S1, Ref. s1). For practical reasons, the progenitor cells employed in the current pilot assay were heterologous (i.e., did not come from the same patient as the wound skin fragments, and were derived from subjects unrelated to the skin donors). Obviously, sweat gland-derived autologous nestin^+^-SGSCs from the same patient whose wounded skin is cultured (which were unavailable to us) may also be tested in this assay system.

## Results

**Fig. 1:**
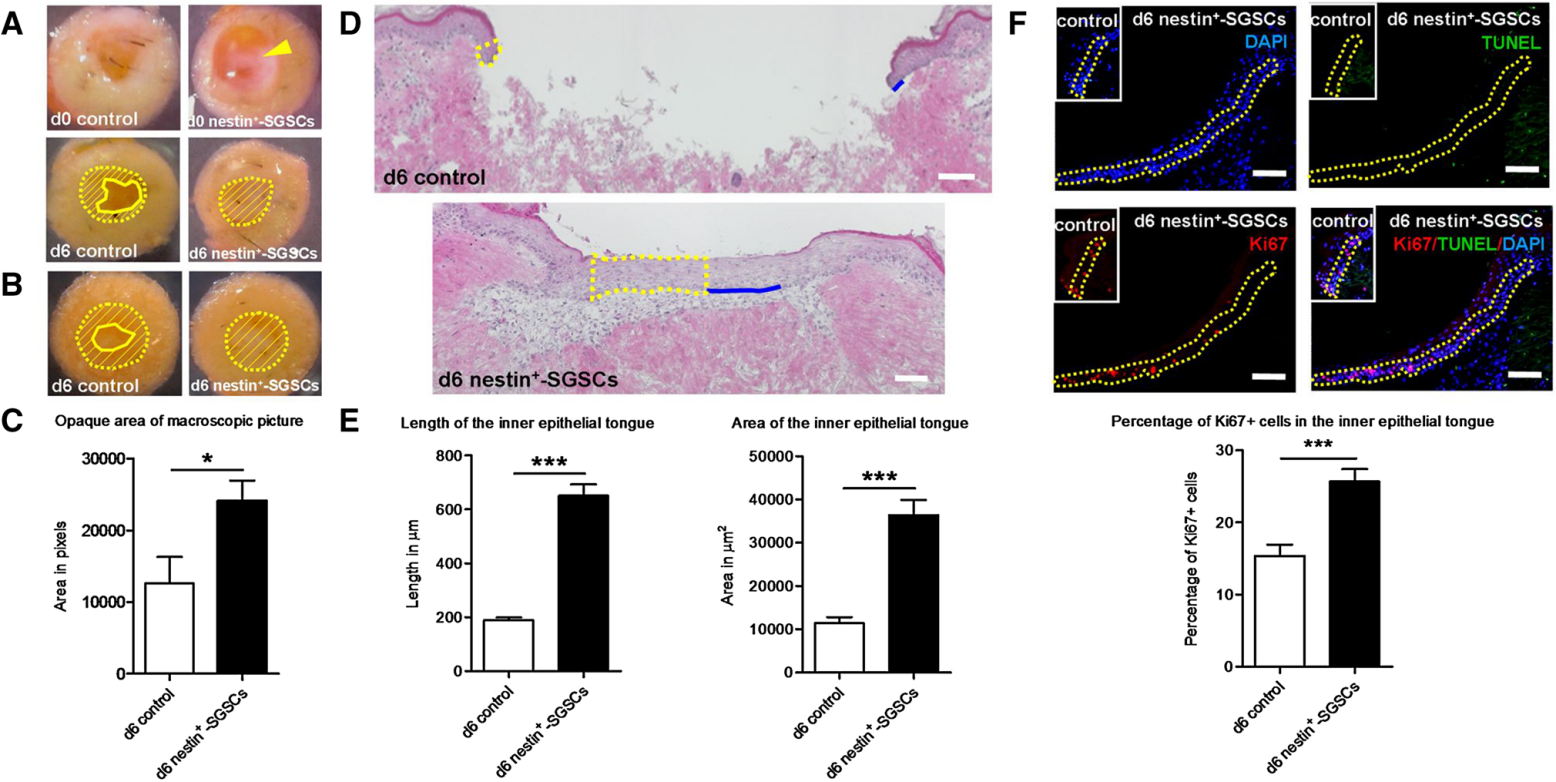
Transplanted human sweat gland stroma-derived nestin^+^ cells (nestin^+^-SGSCs) promote wound reepithelialisation and proliferation of newly generated inner epithelial tongue. **a** Macroscopic pictures of human adult skin punch-within-a-punch biopsies of test individual #2 at different time points. At day 0, in vitro-enriched nestin^+^-SGSCs suspension (yellow arrow) was applied within the inner punch of skin fragment (control group: without nestin^+^-SGSCs; additional controls: see Fig. S3). After 6 days, a thin white-colored layer (opaque) formed. **b** The opaque reepithelialisation area of the inner punch was also visible in the skin fragments of test individual #3 at day 6. (Yellow dotted lines represent the inner wound edges covering the central wound bed, yellow lines mark the reepithelialisation border, yellow diagonal lines represent the area of reepithelialisation which was measured.). **c** The opaque areas in the wounded skin fragments of test individual #2 and #3 were measured and quantified (pixel analysis). Number of independent experiments: *n* = 2 patients (in total, 4 skin punches were analyzed per test/control group, derived from two distinct individuals). **d** Hematoxylin and eosin histochemistry: overview of wounded human skin fragments with or without transplanted nestin^+^-SGSCs at day 6 (yellow dotted lines: area of the inner epithelial tongue, blue lines: length of the inner epithelial tongue). **e** Quantitative analysis of the area and length of the newly generated inner epithelial tongue of skin punch biopsies. **f** Immunofluorescence of Ki67+ (red; proliferation) /TUNEL+ (green; apoptosis) cells in the newly formed inner epithelial tongue at day 6. Ki67 + or TUNEL + cells were counted in the basal cells layer and three or four suprabasal cell layers within the newly formed inner epithelial tongue (yellow dotted lines encircle the reference area) and divided by the number of total cell nuclei (DAPI+) in the reference area. DAPI, 4′-6-diamidino-2-phenylindole; TUNEL, terminal deoxynucleotidyl transferase dUTP nick end labelling. *d* day. Scale bars = 100 µm. **e, f** Number of independent experiments: *n* = 3 patients (in total, 12–15 sections were analyzed per test/control group, derived from three distinct individuals; data were pooled since the results trends in all three independent experiments were comparable). Student *t* test, mean +/− SEM. *p < 0.05; ***p < 0.001

In our first, exploratory pilot assay, 6 days after transplantation of nestin^+^-SGSCs, epidermal regeneration appeared macroscopically to be substantially enhanced in the test group (Fig. S2). Enhanced reepithelialisation was confirmed and quantitatively assessed in two subsequent assays performed with human skin from two different individuals (Fig. 1a-c).

Control assays, run in parallel, demonstrated that in contrast to transplanted adult human nestin^+^-SGSCs, neither extracellular matrix components (Matriderm^®^) nor transplanted human epidermal keratinocytes significantly enhanced human skin reepithelialisation ex vivo (Fig. S3). Keratinocytes were used for comparison given their pivotal role in reepithelialisation and to examine the effects of nestin^+^-SGSCs on keratinocytes during wound healing. These results are in contrast to the accelerated ex vivo wound healing induced by keratinocytes reported by Moll et al. [13], who had used fetal bovine serum. In contrast, we selected a serum-free system, chosen specifically to avoid any confounding effects resulting from components of serum from a different species. Having found no significant effect of human epidermal keratinocytes on reepithelialisation, subsequent experiments compared only wounded human skin fragments with and without transplantation of nestin^+^-SGSCs (5 × 10^4^ positive cells per wound bed topically applied in culture medium) over a culture period of 6 days, i.e., controls were treated only with supplemented serum-free medium [11].

Next, we assessed skin reepithelialisation by quantifying the area and length of the newly generated epithelium (“epithelial tongue”) of the inner wound edges, two key reepithelialisation parameters in wound healing research [13]. Based on the analysis of independent organ culture experiments with wounded human skin fragments from two different individuals, nestin^+^-SGSCs significantly enhanced both the area and the length of the newly formed epithelial tongue of the inner wound edges 6 days after transplantation (Fig. 1d, e). As epithelial tongue length is an indicator of keratinocyte migration during reepithelialisation [12] this suggests that nestin^+^ cell transplantation promotes this process. Furthermore, keratin 5 protein immunoreactivity, a basal layer marker for proliferating, as yet not terminally differentiated keratinocytes, was expressed in the basal and suprabasal layers of the regenerated epithelial tongue 6 days after nestin^+^-SGSCs transplantation (Fig. S4a).

While nestin^+^-SGSCs transplantation did not significantly affect the number of apoptotic (TUNEL+) cells in the regenerated epithelial tongue of the inner wound edges (quantitative data not shown). Instead, the number of proliferating (Ki-67+) cells in the newly generated epithelial tongue of the inner wound edges was significantly increased at day 6 after nestin^+^-SGSCs transplantation (Fig. 1f).

**Fig. 2:**
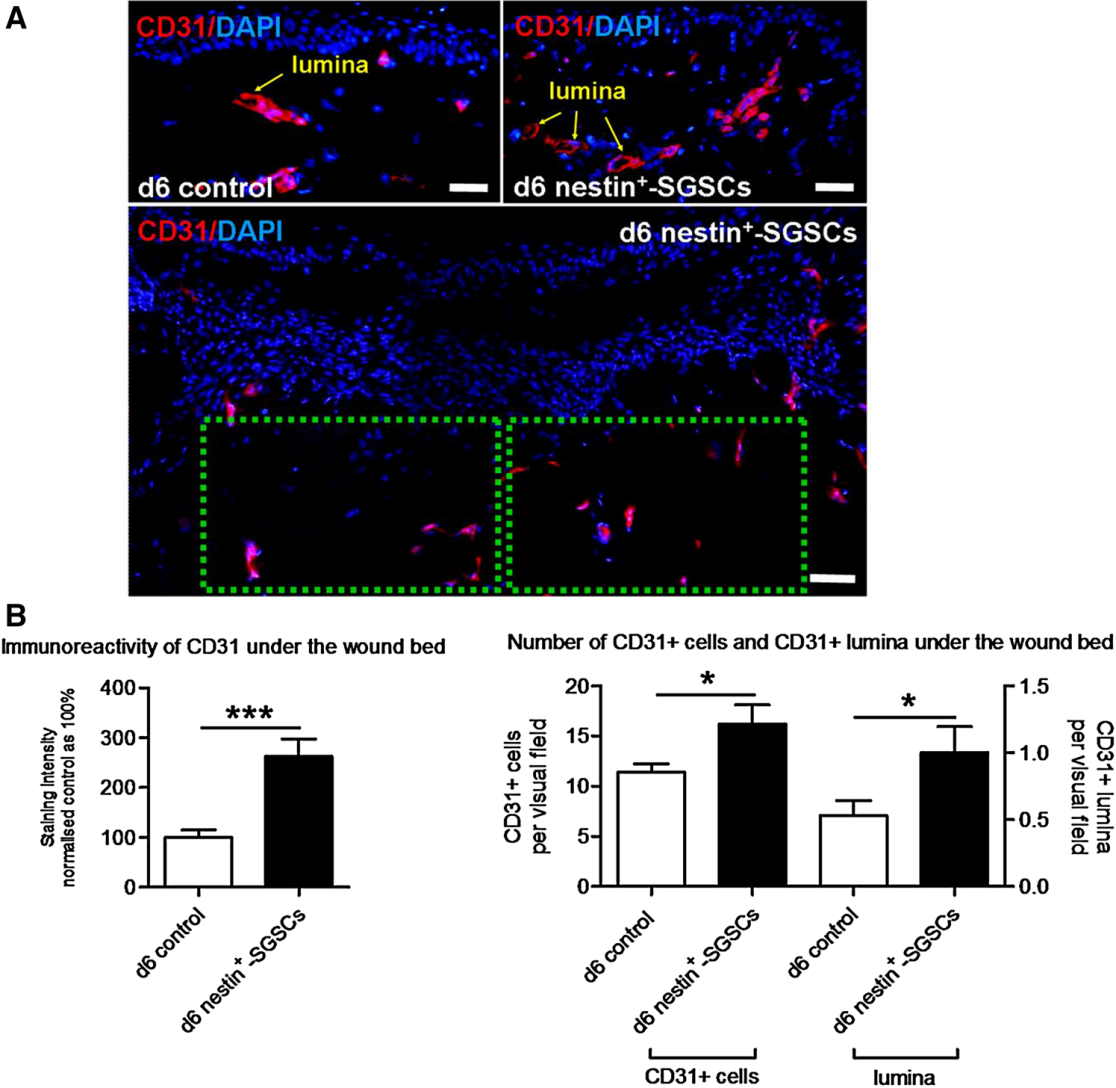
Transplanted human sweat gland stroma-derived nestin^+^ cells (nestin^+^-SGSCs) may stimulate angiogenesis in situ. **a** To analyze angiogenesis under the wound bed, the number of CD31+ cells (red) and of CD31+ blood vessel cross-sections (indicated as lumina; yellow arrow) per visual field was counted at high magnification (×400) by immunofluorescence microscopy in the area under the wound bed (green dotted rectangles; area of wound bed defined according to the presence of nanoparticle-labelled nestin^+^-SGSCs on the adjacent section which was mounted with Vector Mounting Medium with DAPI and was viewed immediately in the rhodamine channel using confocal microscopy, see Fig. S4b, c) (at least 12 visual fields per punch-within-a-punch biopsy were evaluated in total). In addition, the intensity of CD31 immunoreactivity (red) was measured in the reference area. **b** Intensity of the immunoreactivity of CD31, number of CD31+ cells and number of CD31+ lumina was significantly enhanced under the wound bed of test group at day 6 compared to control group. DAPI, 4′-6-diamidino-2-phenylindole. *d* day. Scale bars = 100 µm. Number of independent experiments: *n* = 3 patients (in total, 12–15 sections were analyzed per test/control group, derived from three distinct individuals; data pooled since the results trends in all three independent experiments were comparable). Student *t* test, mean +/− SEM. *p < 0.05; ***p < 0.001

Finally, we tested whether angiogenesis was affected by the transplanted nestin^+^-SGSCs. To do so, we assessed the intensity of the immunoreactivity for the endothelial cell marker CD31 (platelet endothelial cell adhesion molecule, PECAM-1), the number of CD31+ cells, and the number of CD31+ blood vessel cross-sections (lumina) in defined reference areas under the wound bed by quantitative immunohistomorphometry. This revealed that 6 days after experimental skin wounding and transplantation of nestin^+^-SGSCs all three angiogenesis-related parameters were significantly upregulated (Fig. 2a, b).

## Discussion

Despite their limitations and preliminary nature, the preclinical pilot assay data reported here strongly suggest that cell-based therapy with human sweat gland stroma cells greatly enriched for adult nestin^+^ progenitor cells has the capacity to promote both the reepithelialisation and angiogenesis of wounded human skin. Full proof-of-concept will require that these findings can be reproduced with primary nestin^+^ cells from different individuals, in additional skin wound ex vivo assays, and with additional desirable controls (e.g., dermal fibroblasts). Indeed, further studies would be well advised to determine whether the wound healing-promoting effects are dependent on the number of nestin^+^-SGSCs applied to the wound bed, and whether the model is sensitive enough to address any cell density-dependent effects. Moreover, we cannot exclude that the nanoparticles per se exerted some influence on the measured wound healing parameters. That nestin^+^ cell-enriched stromal cells, rather than 100% purified nestin^+^ cells, were used and shown to exert wound healing promoting effects, suggests it may well be dispensable to use highly purified autologous cell preparations. In fact, one wonders whether the presence of other supporting stromal cells may actually facilitate the wound healing-promoting activities of transplanted progenitor cells.

Furthermore, the relatively short duration of skin organ culture (6 days) should be borne in mind. Since the cutaneous architecture remains intact up to and including day 6 [12], the model is ideally placed to study the early phase of wound healing. It remains unclear how long the nestin^+^ cells remain viable in prolonged skin organ culture. However, it would be interesting to determine whether the tissue degeneration seen in long-term organ culture is ameliorated by the addition of nestin^+^ cells (if so, this would encourage one to follow-up whether nestin^+^ cells also exert tissue preserving/anti-aging effects).

Another potentially important factor was the use of nestin^+^ cells derived from heterogenous donors. Whilst it is interesting to observe the wound healing-promoting effects despite the nestin^+^ cells being derived from different donors, for the model to gain translational value it would be useful to determine the effects of nestin^+^ cells on wound healing in ex vivo skin fragments derived from the same patient.

While our pilot study suggests that transplanted nestin^+^ cells stimulate keratinocyte migration, the underlying mechanism is unclear. However, given that mesenchymal stem cells are well known for their extensive repertoire of secretory activities [8, 19, 20], it is conceivable that nestin^+^  progenitor cells, which may be comparable in their highly plastic differentiation potential to adipose-derived mesenchymal stem cells, also impact on keratinocyte migration by secreting migration-enhancing growth factors. Clearly, future studies will have to determine how nestin^+^ cells influence the balance between keratinocyte proliferation, apoptosis, migration and differentiation, culminating in the acceleration of epidermal repair.

We show in the current study that the standardized, serum-free organ-cultured, experimentally wounded full-thickness human skin organ culture model used here provides a simple and instructive, clinically relevant test system for probing novel regenerative medicine strategies, including cell-based wound healing treatment strategies, which complements previous human skin wound healing assays [7, 13, 17, 21]. Not only epidermal regeneration and proliferation, but even angiogenesis can be instructively studied in situ under these organ culture conditions, despite the absence of normal perfusion and the collapse of blood vessels. Besides nestin^+^-SGSCs, other potentially regeneration-promoting skin-derived stem/progenitor cell populations such as CK15+ human hair follicle epithelial progenitors, hair follicle-associated nestin^+^ progenitor cells (Ref. s2-9), and mesenchymal stem cells derived from umbilical cord blood, bone marrow or adipose tissue (Ref. s10-11), can all be tested in this model.

The results of this pilot study should be considered as preliminary and the study has several limitations. For example, one would like to see it repeated (1) with primary nestin^+^ cells from different individuals, (2) in several skin wound ex vivo assays, and (3) with additional desirable controls including, before the findings reported here can be accepted as definitive proof-of-concept. However, our pilot data encourage the systematic pursuit of this promising line of work.

The underlying mechanisms of action, e.g., release of wound healing-promoting growth factors by nestin^+^ cells, direct impact of nestin^+^-SGSCs on the differentiation/proliferation/migration of keratinocytes and endothelial cells, and the stimulation of resident skin progenitor cell populations to undergo differentiation, e.g., into keratinocytes or endothelial cells, all remain to be clarified.

## Conclusions

The current pilot data strongly suggest that adult human progenitor cell populations derived from normal human sweat gland stroma, which are greatly enriched in nestin^+^ cells and which can be isolated with relative ease from human skin [15], stimulate both epidermal regeneration and angiogenesis in wounded human skin ex vivo. This encourages one to further explore the use of adult human nestin^+^-SGSCs in the therapeutic promotion of human skin repair.

## Electronic supplementary material

Below is the link to the electronic supplementary material.

Supplementary material 1 (PDF 7559 KB)

Supplementary material 2 (PDF 89 KB)

## References

[1] Agabalyan NA, Rosin NL, Rahmani W, Biernaskie J (2017). Hair follicle dermal stem cells and skin-derived precursor cells: exciting tools for endogenous and exogenous therapies. Exp Dermatol.

[2] Amoh Y, Li L, Campillo R, Kawahara K, Katsuoka K, Penman S, Hoffman RM (2005). Implanted hair follicle stem cells form Schwann cells that support repair of severed peripheral nerves. Proc Natl Acad Sci USA.

[3] Amoh Y, Li L, Katsuoka K, Hoffman RM (2008). Multipotent hair follicle stem cells promote repair of spinal cord injury and recovery of walking function. Cell Cycle.

[4] Amoh Y, Li L, Katsuoka K, Penman S, Hoffman RM (2005). Multipotent nestin-positive, keratin-negative hair-follicle bulge stem cells can form neurons. Proc Natl Acad Sci USA.

[5] Danner S, Kremer M, Petschnik AE, Nagel S, Zhang Z, Hopfner U, Reckhenrich AK, Weber C, Schenck TL, Becker T, Kruse C, Machens HG, Egana JT (2012). The use of human sweat gland-derived stem cells for enhancing vascularization during dermal regeneration. J Invest Dermatol.

[6] Eming SA, Tomic-Canic M (2017). Updates in wound healing: Mechanisms and translation. Exp Dermatol.

[7] Glinos GD, Verne SH, Aldahan AS, Liang L, Nouri K, Elliot S, Glassberg M, Cabrera DeBuc D, Koru-Sengul T, Tomic-Canic M, Pastar I (2017). Optical coherence tomography for assessment of epithelialization in a human ex vivo wound model. Wound Repair Regen.

[8] Kalinina N, Kharlampieva D, Loguinova M, Butenko I, Pobeguts O, Efimenko A, Ageeva L, Sharonov G, Ischenko D, Alekseev D, Grigorieva O, Sysoeva V, Rubina K, Lazarev V, Govorun V (2015). Characterization of secretomes provides evidence for adipose-derived mesenchymal stromal cells subtypes. Stem Cell Res Ther.

[9] Kruse C, Bodo E, Petschnik AE, Danner S, Tiede S, Paus R (2006). Towards the development of a pragmatic technique for isolating and differentiating nestin-positive cells from human scalp skin into neuronal and glial cell populations: generating neurons from human skin?. Exp Dermatol.

[10] Lau K, Paus R, Tiede S, Day P, Bayat A (2009). Exploring the role of stem cells in cutaneous wound healing. Exp Dermatol.

[11] Lu Z, Hasse S, Bodo E, Rose C, Funk W, Paus R (2007). Towards the development of a simplified long-term organ culture method for human scalp skin and its appendages under serum-free conditions. Exp Dermatol.

[12] Meier NT, Haslam IS, Pattwell DM, Zhang GY, Emelianov V, Paredes R, Debus S, Augustin M, Funk W, Amaya E, Kloepper JE, Hardman MJ, Paus R (2013). Thyrotropin-releasing hormone (TRH) promotes wound re-epithelialisation in frog and human skin. PLoS One.

[13] Moll I, Houdek P, Schmidt H, Moll R (1998). Characterization of epidermal wound healing in a human skin organ culture model: acceleration by transplanted keratinocytes. J Invest Dermatol.

[14] Nagel S, Rohr F, Weber C, Kier J, Siemers F, Kruse C, Danner S, Brandenburger M, Matthiessen AE (2013). Multipotent nestin-positive stem cells reside in the stroma of human eccrine and apocrine sweat glands and can be propagated robustly in vitro. PLoS One.

[15] Petschnik AE, Klatte JE, Evers LH, Kruse C, Paus R, Danner S (2010). Phenotypic indications that human sweat glands are a rich source of nestin-positive stem cell populations. Br J Dermatol.

[16] Sotomayor S, Pascual G, Blanc-Guillemaud V, Mesa-Ciller C, Garcia-Honduvilla N, Cifuentes A, Bujan J (2017). Effects of a novel NADPH oxidase inhibitor (S42909) on wound healing in an experimental ischemic excisional skin model. Exp Dermatol.

[17] Stojadinovic O, Tomic-Canic M (2013). Human ex vivo wound healing model. Methods Mol Biol.

[18] Tiede S, Kloepper J, Ernst N (2009). Nestin in human skin: exclusive expression in intramesenchymal skin compartments and regultaion by leptin. Journal of Investigative Dermatolog.

[19] Won CH, Park GH, Wu X, Tran TN, Park KY, Park BS, Kim DY, Kwon O, Kim KH (2017). The basic mechanism of hair growth stimulation by adipose-derived stem cells and their secretory factors. Curr Stem Cell Res Ther.

[20] Wu M, Ji S, Xiao S, Kong Z, Fang H, Zhang Y, Ji K, Zheng Y, Liu H, Xia Z (2015). JAM-A promotes wound healing by enhancing both homing and secretory activities of mesenchymal stem cells. Clin Sci (Lond).

[21] Xu W, Jong Hong S, Jia S, Zhao Y, Galiano RD, Mustoe TA (2012). Application of a partial-thickness human ex vivo skin culture model in cutaneous wound healing study. Lab Invest.

