# Supplementary material for: Nestin+ progenitor cells isolated from adult human sweat gland stroma promote reepithelialisation and may stimulate angiogenesis in wounded human skin ex vivo
Source: Arch Dermatol Res. 2019 Feb 23;311(4):325–30. doi: 10.1007/s00403-019-01889-x (PMC6469855; doi:10.1007/s00403-019-01889-x)
Supplement: Supplementary file 1 — Supplementary material 1 (PDF 7559 KB) [file 403_2019_1889_MOESM1_ESM.pdf]

Nestin+ progenitor cells isolated from adult human sweat gland stroma promote reepithelialisation and can stimulate angiogenesis in wounded human skin ex vivo

Archives of Dermatological Research

Tian Liao[1*], Janin Lehmann[2*], Sabine Sternstein[3&], Arzu Yay[4&], Guoyou Zhang[5], Anna Emilia Matthießen[6], Sandra Schumann[6], Frank Siemers[7], Charli Kruse[6], Jennifer E. Hundt[8], Ewan A. Langan[8,9§], Stephan Tiede[10§], Ralf Paus[2,9,11@§]

[1]Department of Head and Neck Surgery, Fudan University Shanghai Cancer Center; Department of Oncology, Shanghai Medical College, Fudan University, Shanghai 200032, China.
[2]Monasterium Laboratory, Muenster, Germany
[3]Academic Management, German Sport University of Cologne, Cologne, Germany
[4]Department of Histology and Embryology, University of Erciyes, Kayseri, Turkey
[5]Department of Plastic and Reconstructive Surgery, Shanghai Ninth People's Hospital, Shanghai Jiao Tong University School of Medicine, Shanghai, 200011, China
[6]Fraunhofer Research Institution for Marine Biotechnology and Cell Technology (EMB), Luebeck, Germany
[7]Department of Plastic and Hand Surgery, BG Klinikum Bergmannstrost, Halle, Germany
[8]Department of Dermatology, University of Luebeck, Luebeck, Germany
[9]Centre for Dermatology Research, University of Manchester, and NIHR Manchester Biomedical Research Centre, Manchester, UK
[10]Department of Biochemistry, Children's Hospital, University Medical Center Hamburg-Eppendorf, Hamburg, Germany.
[11]Department of Dermatology and Cutaneous Surgery, University of Miami Miller School of Medicine, Miami, FL, USA

**, &, § contributed equally*

@corresponding author: Ralf Paus, M.D., Dept. of Dermatology & Cutaneous Surgery, University of Miami Miller School of Medicine, Miami, FL, . Email: rxp790@miami.edu

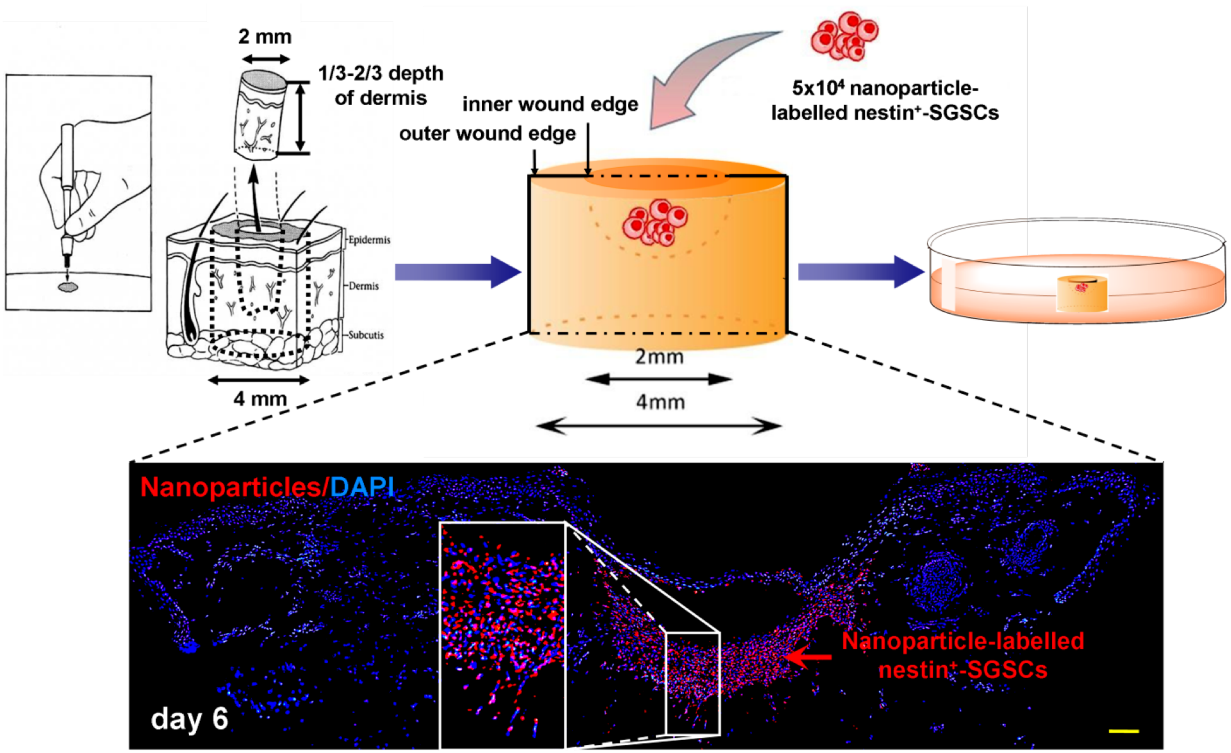

Supplementary figure 1.

Fig. S1. Schematic illustration of the “punch-within-a-punch” human skin wound healing model with transplanted nanoparticle-labelled human sweat gland stroma derived nestin+ cells (nestin^{+}-SGSCs).
Adult human skin, derived from facelift surgery, was used to generate “punch-within-a-punch” biopsies. A 4 mm diameter full-thickness skin punch, consisting of epidermis, dermis and the upper layers of subcutaneous adipose tissue, was generated, from which a 1/3-2/3 depth of 2 mm diameter “cup-like” central portion was vertically removed to create a central punch within a punch. $5x10^{4}$ nanoparticle-labelled nestin^{+}-SGSCs (80% purity) were pipetted into the central wound punch. All skin fragments were cultured in William’s E culture medium (supplemented with 1% penicillin G/streptomycin, 10 mg/ml insulin, 0.05 mg/ml hydrocortisone and 2 mM L-glutamine), which was changed every two days. The epidermis of wounded skin fragments remained constantly exposed to the air. After 6 days, skin fragments were embedded in cryomatrix (OCT), shock-frozen in liquid nitrogen and stored at -80°C until use.
Longitudinal 7 µm cryosections were prepared for nuclear DAPI-staining (blue) and immunofluorescence analysis. The external nestin^{+}-SGSCs labelled with nanoparticle microdroplets were integrated into the granulation tissue formed in the wound bed at day 6 after transplantation. DAPI, 4’-6-diamidino-2-phenylindole. Scale bar = 100 µm. The skin punch biopsy in the upper left of this illustration was modified from "Skin Biopsy (Figure 17.1)." The Patient's Guide to Medical Tests. Houghton Mifflin Company, 1997. http://www.answers.com/topic/skin-biopsy.

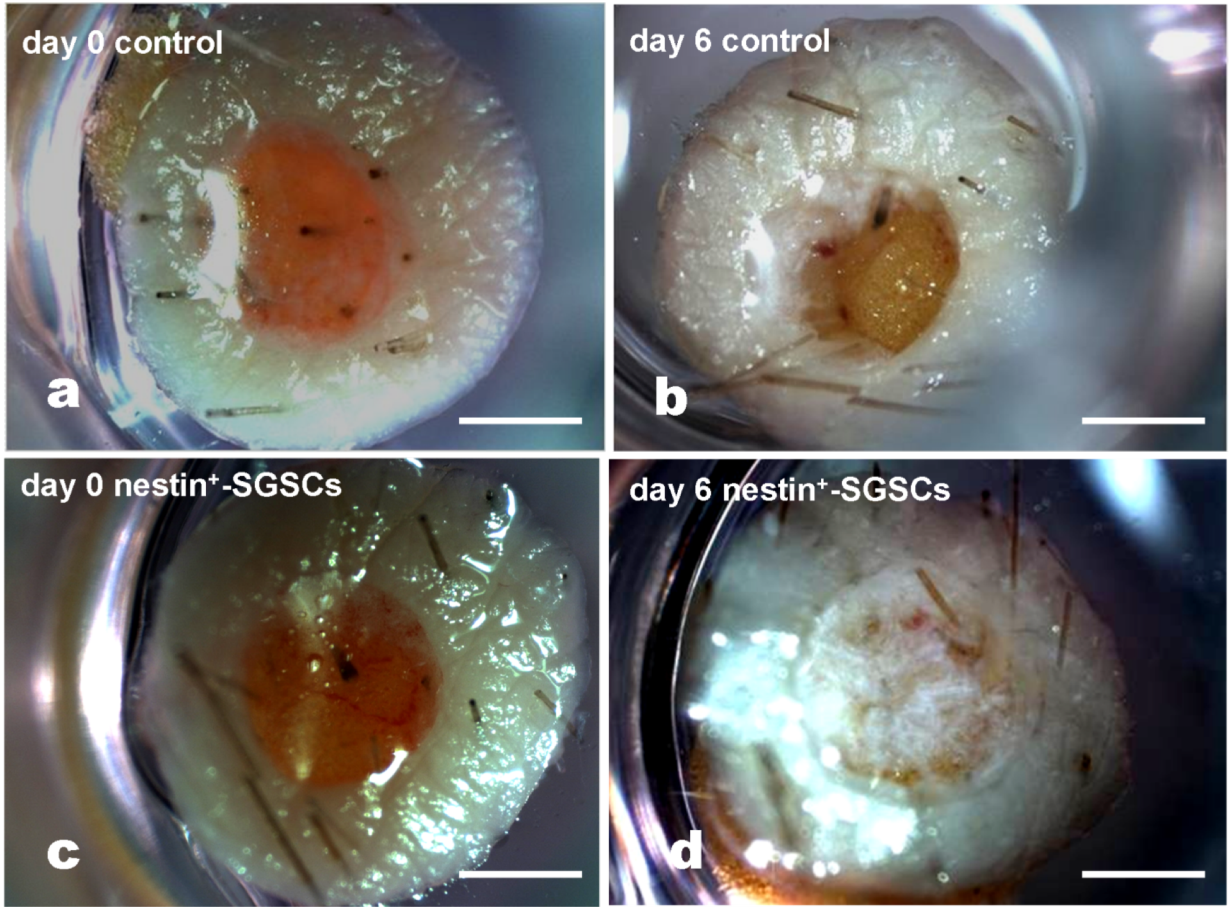

Supplementary figure 2.

Fig. S2. Macroscopic appearance of wounded human skin punches at different time points (individual #1) without (**a, b**) and with (**c, d**) human sweat gland stroma derived nestin$^+$ cells (nestin$^+$-SGSCs) transplantation, photos were taken at day 0 and day 6. Scale bars = 1 mm.

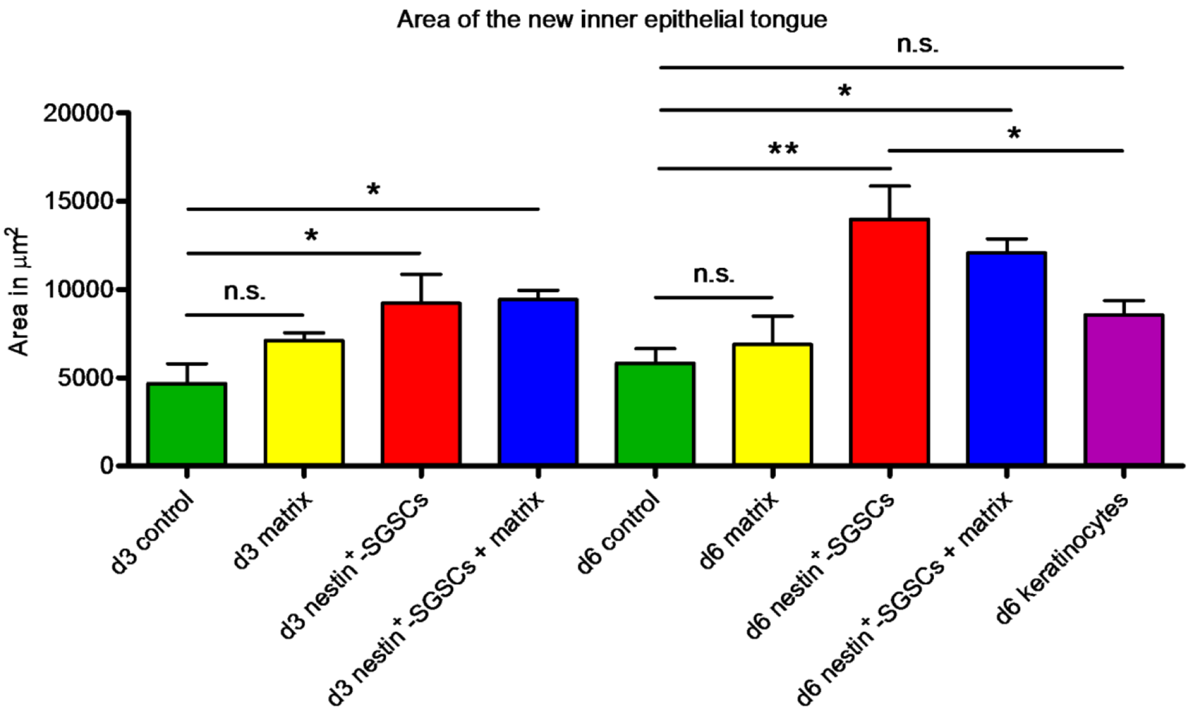

Supplementary figure 3.

Fig. S3. Quantitative analysis of the area of newly generated inner epithelial tongue. Initially, several different test and control conditions were compared with each other. Either extracellular matrix components (matrix: Matriderm®), human sweat gland stroma derived nestin$^+$ cells (nestin$^+$-SGSCs), Matriderm® together with nestin$^+$-SGSCs, or primary human epidermal keratinocytes were transplanted into the inner wound punch (cultured for 3 or 6 days). d: "day". Skin organ culture results from one patient (2-3 skin punches analyzed per test/control group). One-way ANOVA with post hoc Bonferroni comparison, mean +/- SEM. *p<0.05; **p<0.01.

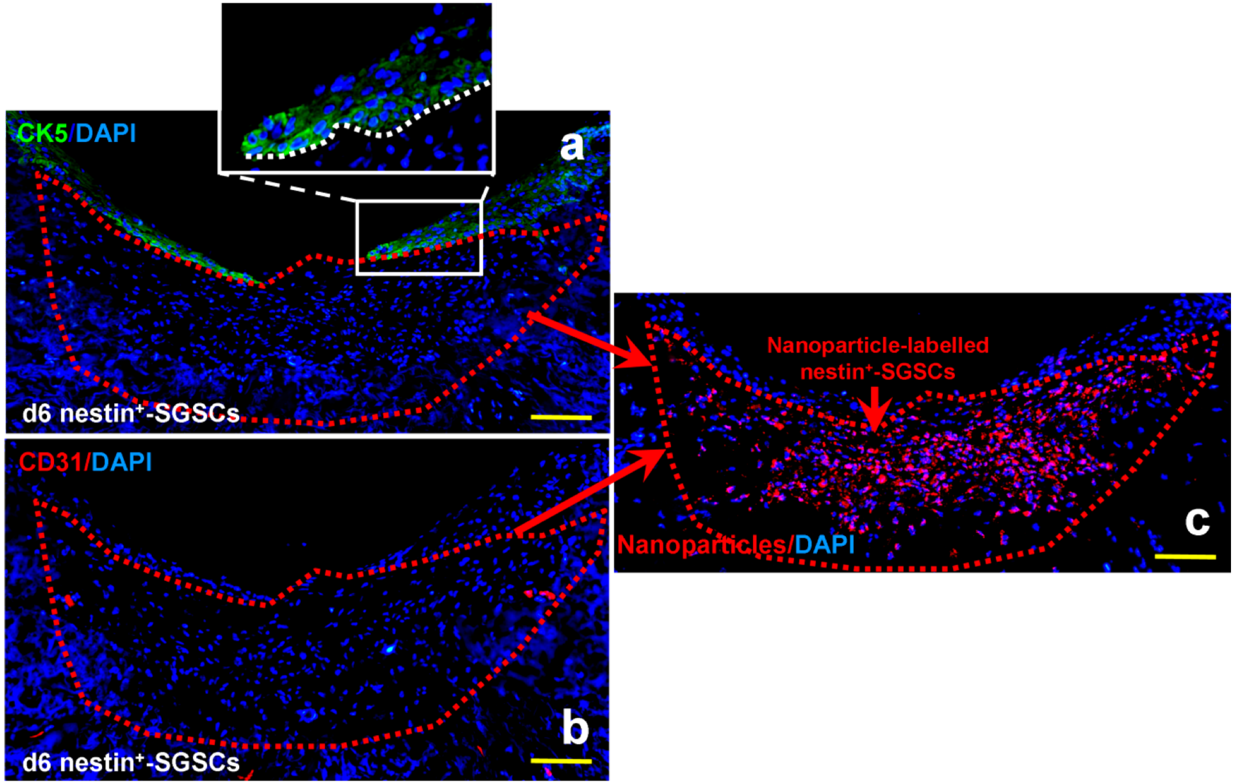

Supplementary figure 4.

Fig. S4. Keratin 5 expression in the newly formed inner epithelial tongue (a) and CD31 staining (b) at day 6. Human sweat gland stroma derived nestin^{+} cells (nestin^{+}-SGSCs) are labelled with nanoparticles (c). a)-c) represent adjacent skin sections (red dotted lines encircle the transplanted nestin+-SGSCs). d: “day”. Scale bars = 100 µm.
