# Supplementary material for: Nestin+ progenitor cells isolated from adult human sweat gland stroma promote reepithelialisation and may stimulate angiogenesis in wounded human skin ex vivo
Source: Arch Dermatol Res. 2019 Feb 23;311(4):325–30. doi: 10.1007/s00403-019-01889-x (PMC6469855; doi:10.1007/s00403-019-01889-x)
Supplement: Supplementary file 2 — Supplementary material 2 (PDF 89 KB) [file 403_2019_1889_MOESM2_ESM.pdf]

Nestin+ progenitor cells isolated from adult human sweat gland stroma promote reepithelialisation and can stimulate angiogenesis in wounded human skin ex vivo

Archives of Dermatological Research

Tian Liao[1*], Janin Lehmann[2*], Sabine Sternstein[3&], Arzu Yay[4&], Guoyou Zhang[5], Anna Emilia Matthießen[6], Sandra Schumann[6], Frank Siemers[7], Charli Kruse[6], Jennifer E. Hundt[8], Ewan A. Langan[8,9§], Stephan Tiede[10§], Ralf Paus[2,9,11@§]

[1]Department of Head and Neck Surgery, Fudan University Shanghai Cancer Center; Department of Oncology, Shanghai Medical College, Fudan University, Shanghai 200032, China.

[2]Monasterium Laboratory, Muenster, Germany

[3]Academic Management, German Sport University of Cologne, Cologne, Germany

[4]Department of Histology and Embryology, University of Erciyes, Kayseri, Turkey

[5]Department of Plastic and Reconstructive Surgery, Shanghai Ninth People's Hospital, Shanghai Jiao Tong University School of Medicine, Shanghai, 200011, China

[6]Fraunhofer Research Institution for Marine Biotechnology and Cell Technology (EMB), Luebeck, Germany

[7]Department of Plastic and Hand Surgery, BG Klinikum Bergmannstrost, Halle, Germany

[8]Department of Dermatology, University of Luebeck, Luebeck, Germany

[9]Centre for Dermatology Research, University of Manchester, and NIHR Manchester Biomedical Research Centre, Manchester, UK

[10]Department of Biochemistry, Children's Hospital, University Medical Center Hamburg-Eppendorf, Hamburg, Germany.

[11]Department of Dermatology and Cutaneous Surgery, University of Miami Miller School of Medicine, Miami, FL, USA

*, &, § *contributed equally*

@corresponding author: Ralf Paus, M.D., Dept. of Dermatology & Cutaneous Surgery, University of Miami Miller School of Medicine, Miami, FL, . Email: rxp790@miami.edu

SUPPLEMENT

Supplementary references

(s1) Coto-Garcia AM, Sotelo-Gonzalez E, Fernandez-Arguelles MT, Pereiro R, Costa-Fernandez JM, Sanz-Medel A (2011) Nanoparticles as fluorescent labels for optical imaging and sensing in genomics and proteomics. *Anal Bioanal Chem* 399:29-42.

(s2) Biernaskie J, Paris M, Morozova O, Fagan BM, Marra M, Pevny L, et al. (2009) SKPs derive from hair follicle precursors and exhibit properties of adult dermal stem cells. Cell Stem Cell 5:610-623.

(s3) Li L, Mignone J, Yang M, Matic M, Penman S, Enikolopov G, et al. (2003) Nestin expression in hair follicle sheath progenitor cells. Proc Natl Acad Sci U S A 100:9958-9961.

(s4) Sieber-Blum M, Grim M, Hu YF, Szeder V (2004) Pluripotent neural crest stem cells in the adult hair follicle. Dev Dyn 231:258-269.

(s5) Sieber-Blum M, Schnell L, Grim M, Hu YF, Schneider R, Schwab ME (2006) Characterization of epidermal neural crest stem cell (EPI-NCSC) grafts in the lesioned spinal cord. Mol Cell Neurosci 32:67-81.

(s6) Yu H, Fang D, Kumar SM, Li L, Nguyen TK, Acs G, et al. (2006) Isolation of a novel population of multipotent adult stem cells from human hair follicles. Am J Pathol 168:1879-1888.

(s7) Yu H, Kumar SM, Kossenkov AV, Showe L, Xu X (2010) Stem cells with neural crest characteristics derived from the bulge region of cultured human hair follicles. J Invest Dermatol 130:1227-1236.

(s8) Tiede S, Bohm K, Meier N, Funk W, Paus R. (2010) Endocrine controls of primary adult human stem cell biology: thyroid hormones stimulate keratin 15 expression, apoptosis, and

differentiation in human hair follicle epithelial stem cells in situ and in vitro. Eur J Cell Biol. 89:769-77. Erratum in: Eur J Cell Biol. 2013 Feb;92(2):87.

(s9) Uchugonova, A., J. Duong, N. Zhang, K. Konig, and R. M. Hoffman. 2011. 'The bulge area is the origin of nestin-expressing pluripotent stem cells of the hair follicle', J Cell Biochem, 112: 2046-50.

(s10) Wu, Y., R.C. Zhao, and E.E. Tredget, Concise review: bone marrow-derived stem/progenitor cells in cutaneous repair and regeneration. Stem Cells, 2010. **28**(5): p. 905-15.

(s11) Yang, J.A., et al., Potential application of adipose-derived stem cells and their secretory factors to skin: discussion from both clinical and industrial viewpoints. Expert Opin Biol Ther, 2010. **10**(4): p. 495-503.

(s12) Yang, S., et al., Umbilical cord-derived mesenchymal stem cells: strategies, challenges, and potential for cutaneous regeneration. Front Med, 2012. **6**(1): p. 41-7.

(s13) Ansell DM, Kloepper JE, Thomason HA, Paus R, Hardman MJ (2011) Exploring the "hair growth-wound healing connection": anagen phase promotes wound re-epithelialization. *J Invest Dermatol* 131:518-528.

(s13) Mardaryev AN, Meier N, Poterlowicz K, Sharov AA, Sharova TY, Ahmed MI, et al. (2011) Lhx2 differentially regulates Sox9, Tcf4 and Lgr5 in hair follicle stem cells to promote epidermal regeneration after injury. *Development* 138:4843-4852.
